# Army News

**Published:** 1919-04

**Authors:** 


### ARMY NEWS

### Dr. W. H. Hargis Returns.

Dr. W. H. Hargis, who during the war served with the rank of major in the Medical Corps of the army, has returned to San Antonio from France. Dr. Hargis was commissioned in the Reserve Corps in 1900 and was the first San Antonio doctor to answer the call to active service at the beginning of hostilities in 1917.

While in France he was regimental surgeon for the 135th Infantry of the 34th Division and was first stationed at Bordeaux. Later at Bassin Docks he was chief surgeon of a command of 15,000 men.

---

Another Texas boy won laurels in the war for which distinction is just now being conferred. Capt. Frank MacGregor, U. S. A., Medical Corps, was invested with the English Military Cross by King George only a few days ago. Captain MacGregor was one of 500 American medicos assigned to service with the British and was with a regiment of Scot Highlanders. He won the cross by exceptional services at Chateau Thierry, where for ten days he dressed wounds on the open fields. For this he was advanced to the rank of captain.

---

### Major Hal Gambrell.

Major Hal. Gambrell, well known Dallas doctor, has returned home after serving overseas. He returned to Camp Bowie with the 11th ammunition train, which will be demobilized there. Major Gambrell was one of the first Dallas physicians to volunteer when war was declared. He will remain in Dallas for only a few days, having been ordered to report at the base hospital at Atlanta, Ga. While in France Major Gambrell besides being on duty with the ammunition train also served some months in the large army hospitals in Paris.

---

Official information has been received in Sherman of the promotion of a well known Grayson county physician, Dr. Joe Wolfe of Van Alstyne, from a lieutenancy to a captaincy.

---

Word has been received in Whitesboro that Dr. Parrish Acton, who went into the army as first lieutenant in the Medical Corps, has been promoted and is now a captain. His commission as captain dates back to February 17. A few days after his promotion he received the French Croix de Guerre, following on official citation for excellent work at Blanc Mont.

---

Doctor Walter Noble, of Aransas Pass, who has been attached to the base hospital at Camp Travis, has received his discharge and returned home.

---

Friends of Dr. Byron S. Bruce of Dallas have been advised of his promotion to the rank of major in the Medical Corps of the United States Army. Major Bruce is serving with the Thirty-Sixth Division. The division is expected to remain in France several weeks.

---

Major James C. Greenway, chief of the medical service department at the Camp Bowie base hospital, has been discharged from the army after a service of more than eighteen months. He has returned to Yale, where he will resume his old duties as director of the health department.

---

**Sam Herford Staples Poses as Dr. Robert H. Milwee of Dallas and Is Made First Lieutenant.**

March 20.—Sam Herford Staples, said by authorities to be the son of Dr. Tom C. Staples of Wylie, Texas, who obtained a commission as first lieutenant in the Medical Corps of the United States Army under the name of Dr. Robert H. Milwee, a physician of Dallas, Texas, was released by Federal authorities March 19th after being in custody for more than a month. Staples, according to Federal agents, could not be prosecuted for the impersonation of an officer, because his commission was bona fide although under an assumed name; and charges for a fraudulent enlistment could not have been sustained either, they declared.

Federal agents are at a loss to know how Staples passed the army examination for the Medical Corps, having, as he confessed to them, no knowledge of medicine. His first assignment, he says, was at Camp Greenleaf, Ga.

---

First Lieutenant T. P. McLendon, for nine years a physician at Wortham, has moved his family to Corsicana and will in future practice his profession in that city.

Doctor McLendon has just returned from six months service with the army in France. He was with Evacuation Hospital 23. He landed in France on July 28 and has many experiences.

### Dr. Hodde Improving.

Dr. F. H. Hodde of Burton, who has been seriously ill with pneumonia, is reported slightly better, and numerous friends will be glad to learn of his improvement. Dr. L. F. Hodde of Galveston, and Henry Hodde, Jr., of Ansley, Louisiana, are in Burton attending their brother's bedside.

Dr. J. H. Walker has returned from France and is now at his home in Alvord. The Doctor will be welcomed back by his many friends.

### "Made in Germany"—Not Wanted.

There is a soldier at Walter Reed Hospital in Washington who certainly is supporting the boycott of German goods. When he came out of the ether the other day he found a good sized bit of shrapnel tied to a button of his pajama coat.

"What's this?" he asked in a puzzled way.

"We thought you might like it as a souvenir," smiled the Red Cross nurse. "The surgeon took it from your leg."

"Take it away," snapped the doughboy. "I don't want anything around me that was made in Germany."

### Temple Sanitarium Open House.

The Temple Sanitarium has decided to keep "open house" at the Sanitarium in Temple on Friday and Saturday, May 16th and 17th, following the meeting of the Texas State Medical Convention at Waco. This is for the purpose of enabling the physicians and surgeons of the State to visit this well-known Sanitarium after the convention and see just what is being done there, a number of them having suggested that they would like to do so. It will afford a fine opportunity to inspect this big modern hospital and learn at first hand of its work.

Dr. A. W. Morton, the celebrated San Francisco surgeon, has written that he will make a special trip to Texas to visit the Temple Sanitarium, but has not yet given the date of his contemplated visit, though it is hoped he may be there when the Texas doctors visit the Sanitarium, May 16th and 17th.

THE DISTINCTION.—"Pa," asked Willie, "what's the difference between an invalid and a sick person?"

"An invalid, my son," answered pa, "has money."—Judge.

### STATE ITEMS

The Bacteriological Laboratory of G. H. Sherman M. D., Detroit, Mich., Manufacturer of Bacterial Vaccines is in need of a detail man for the State of Texas. In replying give full details as to qualifications, experience, age, salary, etc. Resident of territory mentioned preferred.

---

Wanted at Paige—A good young doctor, one who can handle a good practice. Address communication to D. C. Lea, D. D. S., Paige, Texas.

---

Orders were received from the Surgeon General of the United States Army by Lieut. Col. S. W. French, commanding officer at the base hospital at Camp MacArthur, ordering immediate disbandment of the hospital. Colonel French says two or three months will be required to completely disband the base hospital.

---

Dr. G. A. Spivey, of Dallas, is in receipt of advice from his son, Dr. G. Howard Spivey, who is now convalescent after being gassed on the field of battle in France, saying that he has been promoted to a captaincy in the United States Army Medical Corps.

---

Doctor J. L. Stahl who has just been given his honorable discharge from the medical corps of Uncle Sam's forces, has returned to Gonzales, the home of his boyhood and will hang out his shingle there.

---

A sentence of dismissal from the army and confinement at hard labor for two years imposed by court-martial in Porto Rico upon Capt. Samuel H. Hodgson, Army Medical Corps, has been commuted by President Wilson to a reprimand, the War Department announces. Captain Hodgson was found guilty of having made statements indicating his admiration of the German people and, according to the specifications entered against him of "favoring the cause of Germany."

---

Captain, Stephen D. Bullington, of Dallas, has received his discharge from the U. S. medical corps and has returned to his home to resume his medical practice. Captain Bullington has been stationed at Fort Sam Houston and at the cavalry officers' training school at Camp Stanley.

